# Eight new species of *Strongylophthalmyia* Heller from Vietnam with a key to species from Vietnam and neighbouring countries (Diptera, Strongylophthalmyiidae)

**DOI:** 10.3897/zookeys.625.8711

**Published:** 2016-10-19

**Authors:** Tatiana V. Galinskaya, Anatoly I. Shatalkin

**Affiliations:** 1 Biological Faculty, Entomology Department, Lomonosov Moscow State University, Moscow, Russia, Leninskie gory, 1–12, 119234; 2 Zoological Museum, Lomonosov Moscow State University, Ul. Bol’shaya Nikitskaya 6, Moscow, 125009, Russia

**Keywords:** Dipterous fauna, new species, Strongylophthalmyia, Strongylophthalmyiidae, systematics, Vietnam

## Abstract

Seventeen species of *Strongylophthalmyia* are recognized in the fauna of Vietnam, including eight new species: *Strongylophthalmyia annulipes* **sp. n.**, *Strongylophthalmyia basisterna* **sp. n.**, *Strongylophthalmyia dichroa* **sp. n.**, *Strongylophthalmyia gavryushini* **sp. n.**, *Strongylophthalmyia obtecta* **sp. n.**, *Strongylophthalmyia orchidanthae* **sp. n.**, *Strongylophthalmyia stricta* **sp. n.**, *Strongylophthalmyia tomentosa* **sp. n.** *Strongylophthalmyia angusticollis* Frey, *Strongylophthalmyia fascipennis* Frey, *Strongylophthalmyia metatarsata* Meijere, *Strongylophthalmyia splendida* Yang & Wang, and *Strongylophthalmyia thaii* Papp are recorded for the first time from Vietnam. The male of *Strongylophthalmyia splendida* and female of *Strongylophthalmyia thaii* are described for the first time. A key to 34 species of *Strongylophthalmyia* of the fauna of Vietnam and continental southeast Asia, including the Oriental southern region of China is provided.

## Introduction

The Strongylophthalmyiidae is a small family of acalyptrate Diptera containing two genera, *Strongylophthalmyia* Heller, 1902 and *Nartshukia* Shatalkin, 1993. The genus *Nartshukia* is known only by the single female specimen of *Nartshukia musiva* Shatalkin, 1993 from Vietnam. The genus *Strongylophthalmyia* includes 54 species to date, occurring in the Nearctic (two species), Palaearctic (eight species), Oriental (40 species) and Australasian (9 species) regions (Iwasa and Evenhuis 2014). Two species described from Madagascar by Verbeke (1963, 1968) were transferred to Clusiidae by Barraclough (2000). As our studies show, the Vietnamese fauna of the genus includes 17 species. Eight of them are described in this paper as new, and five of them are reported for Vietnam for the first time.

## Materials and methods

This study is part of an ongoing series of studies on the Vietnamese cyclorrhaphous fauna. A key is composed for species from Vietnam, Burma, Oriental southern Region of China and Thailand; the genus has not yet been recorded from Laos or Cambodia. The specimens of new species of *Strongylophthalmyia* described in this paper are rare in our collection, and the colouration of the abdomen is important for determination; hence, genitalic characters are not explored in the descriptions of new species.

Types of the new species are deposited in the collection of Zoological Museum of Moscow University (ZMUM).

In the key and descriptions of species, morphological terminology, abbreviations of wing veins, and wing cells, are after Cumming and Wood (2009). Measurements are given in millimetres. Labels of specimens are quoted verbatim. Frontal index = the ratio between height of the frons from its anterior margin to hind ocelli and from hind ocelli to vertex or vti.

## Results

The genus *Strongylophthalmyia* includes strikingly elegant flies with elongated bodies and slender legs. These flies have body lengths from 2.3 mm to 7.5 mm (the smallest is *Strongylophthalmyia palpalis* Papp, 2006 the largest is *Strongylophthalmyia gigantica* Iwasa & Evenhuis, 2014).

**Figure 1.**
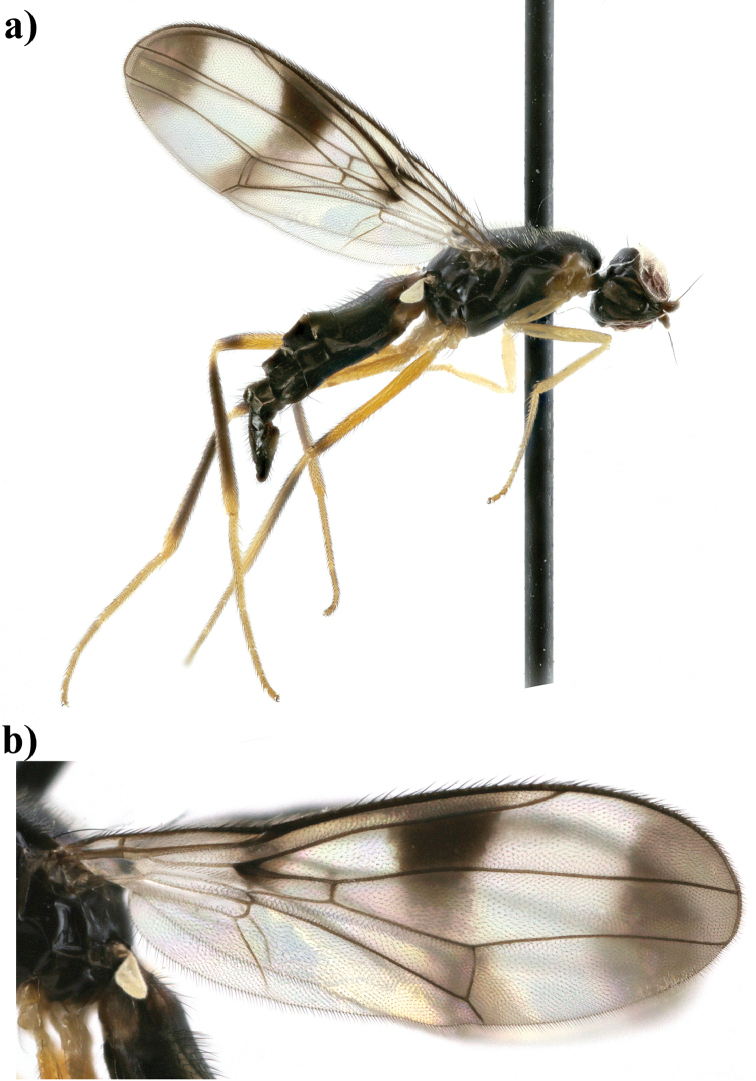
*Strongylophthalmyia annulipes* sp. n. **a** habitus, lateral view **b** wing.

**Head** (Figure 1a) is spherical or extended in profile (extended in *Strongylophthalmyia splendida* Yang & Wang, 1996); the gena is narrow; the facial sclerotization is interrupted by membrane medially; and the ocellar tubercle is moved forward and is often situated in the middle of the frons. Females often have a large bulbous clypeus, but it is smaller and band-like in males. First flagellomere is short, rounded.

Several species of this genus have sexual dimorphism in the antennal structure. The males of some *Strongylophthalmyia* differ in developing of the dorsal process of the first flagellomere (Figure 10d, e).

The arista is usually bare, but several Oriental species have small setulae on the arista. Males of some of these species groups have modified palpi, which can be yellow (compared to the black female palpus), bilobate and with black scales or thick setulae. Chaetotaxy of head: 2–3 orbital setae, usually with three hair-like small frontal setae, one ocellar seta, one inner vertical seta and one outer vertical seta (absent in *Strongylophthalmyia splendida*), one postocellar seta, one paravertical seta (absent in some species), vibrissa absent.

**Figure 2.**
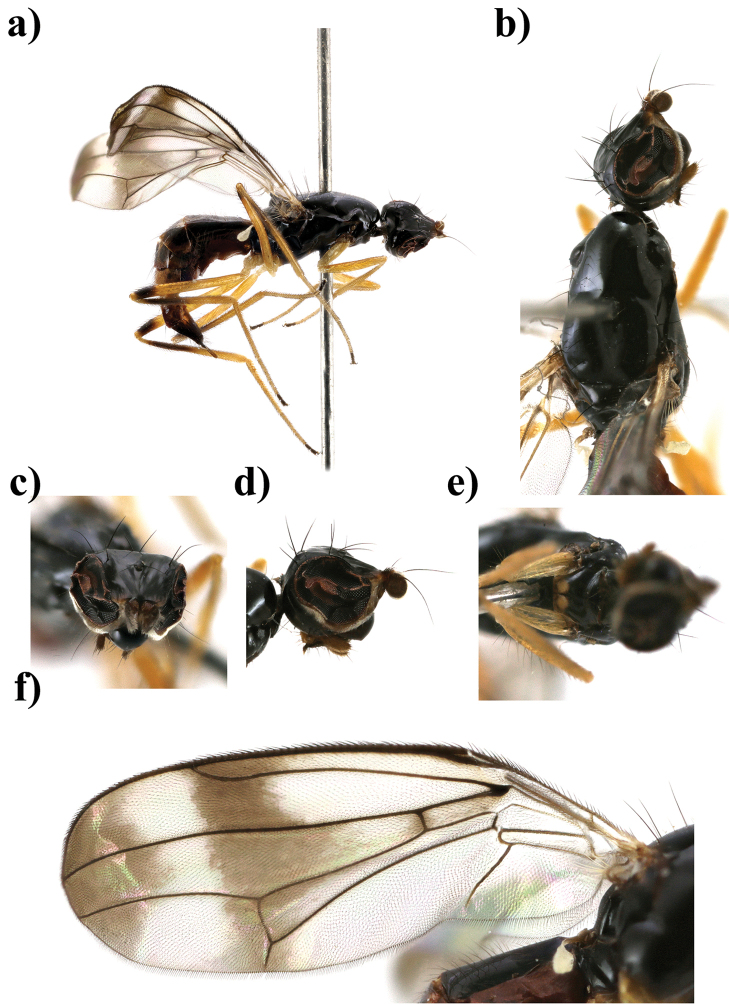
*Strongylophthalmyia basisterna* sp. n. **a** habitus, lateral view **b** head and thorax, dorsal view **c** head, anterior view **d** head, lateral view **e** basisternum **f** wing.

**Thorax** is coloured from yellow (Figure 6) to black (Figure 2b). Several species are characterised by a black thorax, and a postpronotum and propleuron and often basisternum that are entirely or partly yellow. Several species are black with a pair of reddish yellow spots (in females) or one large spot (in males) on the basisternum between the fore coxae (Figure 2e). Finally, two species, described in this paper as new, *Strongylophthalmyia obtecta* sp. n. (Figure 5a,b) and *Strongylophthalmyia stricta* sp. n. (Figure 7a), are black with brownish or yellowish spots on the postpronotum (laterally), and with yellowish spots around the fore spiracles. These two species may be related to *Strongylophthalmyia papuana* Iwasa & Evenhuis, 2014 and *Strongylophthalmyia gigantica* Iwasa & Evenhuis, 2014, both from Papua New Guinea, because these four species are characterized by predominantly black legs. Since the black colouration of legs can fade in preserved specimens, these species appear in multiple parts of the key. The mesonotum is covered with yellowish setulae or it is bare with black setae located in rows.

Chaetotaxy of thorax: 1 anepisternal seta, 2 notopleural setae (one notopleural seta in Nearctic *Strongylophthalmyia pengellyi* Barber, 2006), 1 supraalar seta, 1 postalar seta, 1–2 dorsocentral setae, 1 apical scutellar seta. Postpronotum usually with some setulae, rarely with long setae. *Strongylophthalmyia splendida* has a strong black seta in the anterior part of the mesonotum, near the postpronotum (Figure 9b: marked by arrow), which we consider as sublateral (Hennig 1973: 184, Figure 109).

**Legs** are slender, usually bare. Hind legs of males of some species bear small papillae, having one papilla on trochanter and two papillae on femur (these papillate protuberances are variable; some species with only on femur, others on both trochanter and femur, some with just one, others up to three, some bare, others with long stiff setae), being bubble-like basally and conical (with yellow or black setulae) subbasally. Male fore femur sometimes with short stout spines dorsally (Figure 4a).

**Wings** have a distinct costal break just before merging of costa and subcosta. The subcosta is incomplete, not quite reaching the wing margin (Figure 1a). Transverse vein CuA_2_ forms distal border of anal cell, convex. Veins R_4+5_ and M_1_ are parallel or slightly converging in apical third. Vein A_1_+CuA_2_ is distinctly bent, not reaching wing margin. Wing is transparent, wholly infuscated, with brownish apical spot or with cross-band on dm-cu and short cross-band anteriorly level of Rs. Greater ampulla is absent. Halter is coloured from whitish to yellow, rarely with blackish-grey knob.

**Abdomen** is slender, black, sometimes yellow, its colour patterns in some cases (*Strongylophthalmyia trifasciata* Hennig, 1940) differs in females and males.

The structure of **male genitalia** are known only for several species of *Strongylophthalmyia* (not for *Nartshukia*). Epandrium without surstyli (some authors (Iwasa 1998; Iwasa and Evenhuis 2014) consider that surstyli are completely fused with epandrium). However, genitalia of species of *Strongylophthalmyia crinita* group have appendages similar to surstyli and attributed to this type of lobe (Shatalkin 1995). The hypandrium is well-developed, bearing two pairs of lobes. The origin of these lobes remains unclear (Steyskal 1987, Shatalkin 1995, Iwasa 1998, Barber 2006, Lonsdale 2013, Iwasa and Evenhuis 2014). Phallus and phallapodeme are very long; however, several exceptions are known. The male of *Strongylophthalmyia verrucifera* Shatalkin, 1996 is characterized by a short apodeme of the aedeagus; on the contrary, males of *Strongylophthalmyia paula* Shatalkin, 1993 have truncated aedeagus.

**Female terminalia** have ovipositor quite elongate, slender, with unsclerotized cerci. One or two (in *Strongylophthalmyia pengellyi*) spermathecae present (Iwasa 1998, Barber 2006). Tergite and sternite 7 are fused into syntergosternite.

**Ecology.** Adult flies can be found on leaves of bushes and trees, on stumps, and fallen logs. Larvae of some Holarctic species are characterised by biramous anterior spiracles, and live under the bark of rotting logs of aspen (*Strongylophthalmyia ustulata* (Zetterstedt, 1847), *Strongylophthalmyia pictipes* Frey, 1935, *Strongylophthalmyia angustipennis* Melander, 1920, *Strongylophthalmyia pengellyi*) and under bark of rotting logs of birch and elm (*Strongylophthalmyia stackelbergi* Krivosheina, 1981) (Krivosheina 1981, Barber 2006).

**Notes.** Neal L. Evenhuis in his review kindly noted that there is apparently a serious mistake in Yang and Wang (1998) with regard to the description and wing illustrations of *Strongylophthalmyia splendida* and *Strongylophthalmyia yaoshana*. The descriptions of the wing are either switched or the illustrations are switched. *Strongylophthalmyia splendida* is described as having a hyaline wing, yet the illustration claearly slows bands; whereas *Strongylophthalmyia yaoshana* is described as having a banded wing, yet the illustration clearly shows it to have a hyaline one. As the holotypes of each species are lost (Wang Xin li, pers. comm.; also N.L. Evenhuis could not find them during Wang’s and my search for them when N.L. Evenhuis was in Beijing in 2014; only a paratype of *Strongylophthalmyia bifasciata* could be found) there is no definitive way to determine which species is which.

### Key to species of genus *Strongylophthalmyia* from Vietnam and neighbouring countries (Burma, China, and Thailand)

**Table:** 

1	Mesonotum yellow, usually with pair of brown longitudinal stripes, in one case these longitudinal stripes converging before suture	**2**
–	Mesonotum black, sometimes yellow in anterior part to suture	**5**
2	Mesonotum with black arcuate concave spot anteromedially; mesonotum with pair of narrow black longitudinal stripes behind suture, these stripes continue onto scutellum. Pedicel black. Body length 4.3. Vietnam (Figure 6)	***Strongylophthalmyia orchidanthae* sp. n.**
–	Mesonotum at most with pair of brown longitudinal stripes. Pedicel yellow	**3**
3	Abdomen entirely yellow. Head yellow, except ocellar tubercle. Male: first flagellomere dorsally without very short conical process anterior to base of arista; fore femur without spines	**4**
–	Abdomen black, at most yellow at base. Head partly yellow; frons black, yellow or grey in anterior fourth (female), yellow in anterior third (male). Male: first flagellomere dorsally with very short conical process anterior to base of arista; fore femur without spines. Body length 2.3–3.0. Thailand	***Strongylophthalmyia palpalis* Papp, 2006**
4	Wing with well-developed apical spot and with crossband on dm-cu vein. Mesonotum with pair of weak brownish stripes on each side posteriorly from dorsocentral setae. Body length 3.5–4.7. Thailand	***Strongylophthalmyia dorsocentralis* Papp, 2006**
–	Wing with poorly developed greyish spots. Mesonotum with pair of weak brownish stripes from suture to scutellum. Body length 4.7. Indonesia (Java), Thailand	***Strongylophthalmyia lutea* (Meijere, 1914)**
5	Mesonotum black in posterior part behind suture; yellow in anterior part, sometimes with brown median strip, going from anterior margin of mesonotum. Body length 3.7–5.0. Burma, Vietnam	***Strongylophthalmyia elegantissima* Frey, 1956**
–	Mesonotum entirely black	**6**
6	Postpronotum, propleuron and often basisternum entirely or partly yellow or yellowish-brown	**7**
–	Postpronotum and propleuron totally black	**19**
7	Wing transparent or with pale brownish apical spot	**8**
–	Wing with well-expressed brown cross-band on dm-cu vein and with apical spot	**12**
8	Frons completely black. Veins R_4+5_ and M_1+2_ straight and parallel, not convergent apically	**9**
–	Frons partly yellow. Apical part of vein M_1+2_ strongly curved in direction of vein R_4+5_	**11**
9	Abdomen entirely black. Anepisternum without tuft of silvery setulae medially. Legs yellow, mid and hind femora widely darkened. Male fore femur without dorsal row of black spines and with thick medioventral thorn-like tight cluster of setulae basally. Female unknown. Male body length 3.1. Thailand	***Strongylophthalmyia macrocera* Papp, 2006**
–	Abdomen shining black, basally yellowish. Anepisternum with tuft of silvery setulae medially. Legs yellow, at least hind femur with apical brown ring. Male fore femur with dorsal row of 5–7 short black spines basally (Fig. 10a)	**10**
10	Male first flagellomere dorsally with long and slender process (see Papp et al. 2006, fig. 56; this paper - Fig. 10d). Hind tibia darkened dorsally and ventrally in basal two thirds. Body length 3.4–3.8. Vietnam, Thailand (Figure 10)	***Strongylophthalmyia thaii* Papp, 2006**
–	Male first flagellomere without dorsal process. Hind tibia darkened dorsally and ventrally in basal half. Female unknown. Male body length 4.0. Burma, Thailand	***Strongylophthalmyia spinosa* Frey, 1956**
11	Abdomen entirely black. Frons black, anteriorly yellow. Wing with pale brownish apical spot. Body length 4.5. Burma	***Strongylophthalmyia curvinervis* Frey, 1956**
–	Abdomen black, tergite 6 brownish yellow. Frons yellow with brownish area around ocellar tubercle and on vertex. Wing transparent (Fig. 9a). Body length 3.4. China, Vietnam (Figure 9)	***Strongylophthalmyia splendida* Yang & Wang, 1998**
12	Abdomen mostly matte yellow. Head with 2 orbital setae. Female unknown. Male body length 3.4. Vietnam (Figure 4)	***Strongylophthalmyia gavryushini* sp. n.**
–	Abdomen shining black, at most yellowish basally. Head usually with 3 orbital setae (male and female)	**13**
13	Arista with short setulae	**14**
–	Arista bare	**16**
14	Frons black	**15**
–	Frons black, yellow in anterior third. Basisternum with a pair of yellow spots. Body length 5.5–6.0. Burma.	***Strongylophthalmyia humeralis* Frey, 1956**
15	Wing: median transverse band between C and R_2+3_ undeveloped; apical spot large, extended from beginning of R_2+3_ vein; short cross-band at level of R_s_ undeveloped. Face yellow, arista yellow. Body length 5.0 (male), 6.0 (female). China (Zhejiang)	***Strongylophthalmyia bifasciata* Yang & Wang, 1992**
–	Wing median transverse band between C and R_2+3_ well-developed, reaching costal margin; apical spot small, its length equal distance from this spot to R_2+3_ vein; short cross-band at a level of R_s_ in anterior half of wing developed. Face black, arista dark brown. Male unknown. Female body length 5.0. China (Guangxi)	***Strongylophthalmyia yaoshana* Yang & Wang, 1998**
16	Abdomen black, broadly yellow basally, tergite six with lateral yellow spots extending from anterior margin to two thirds of its length. Wing with two brownish marks: short cross-band at a level of R_s_ undeveloped; median cross-band on a dm-cu vein almost undeveloped between C and R_2+3_. Male unknown. Female body length 3.6. Vietnam (Figure 8)	***Strongylophthalmyia tomentosa* sp. n.**
–	Abdomen entirely black, or yellow basally. Wing with three brownish marks: apical spot, cross-band on dm-cu vein and short crossband at level of R_s_ in anterior half of wing. Median cross-band well developed between C and R_2+3_	**17**
17	At least area around anterior spiracle and postpronotum laterally yellow. Fore legs entirely yellow. Body length 3.8–4.7. Vietnam (Figure 1)	***Strongylophthalmyia annulipes* sp. n.**
–	Area around anterior spiracle and postpronotum laterally brown. Fore coxa and femur partly black	**18**
18	Fore femur black, basally and apically with narrow yellow ring. Fore tibia yellow, brownish in basal third. Two (in males) or three (in females) last segments of tarsi contrastingly black. Hind trochanter of male with posterior round blackish spot; hind femur basally with round posteroventral process, and with small subbasal process bearing patch of yellow setulae situated on the posterior surface of hind femur. Wing with wide median crossband (Fig. 7b). Body length 3.3 (male), 4.8 (female). Vietnam (Figure 7)	***Strongylophthalmyia stricta* sp. n.**
–	Fore femur yellow with blackish spot on apical third; fore tibia entirely yellow; female with 2 last segments of tarsi black (male unknown). Wing with narrow median cross-band (Fig. 5b). Body length 4.2–4.6. Vietnam (Figure 5)	***Strongylophthalmyia obtecta* sp. n.**
19	Hind femur entirely yellow or with apical or subapical dark brown ring	**20**
–	Hind femur largely black, including basal half of segment	**29**
20	Arista with short distinct setulae. Basisternum with pair of reddish yellow spots (in females) or with one large spot (in males) between fore coxae. Body length 5.5–6.5. Burma, Vietnam	***Strongylophthalmyia angusticollis* Frey, 1956**
–	Arista bare. Basisternum with pair of reddish yellow spots or black	**21**
21	Wing at least darkened apically	**22**
–	Wing totally transparent	**25**
22	Frons posteriorly black, anteriorly yellow	**23**
–	Frons entirely black, at most with brownish spot between antenna and eye	**24**
23	All femora with contrasting apical blackish ring. Thorax with 1 dorsocentral seta. Wing apically, r-m and dm-cu darkened. Body length 4.0–4.4. Burma	***Strongylophthalmyia punctum* Frey, 1956**
–	Fore femur without apical blackish ring. Thorax with 5 dorsocentral setae. Male: wing with median cross-band and apical spot; first flagellomere with long tubular dorsal process, covered with black setulae. Female: wing with median cross-band almost undeveloped. Body length 3.4–3.5. Thailand	***Strongylophthalmyia freidbergi* Shatalkin, 1996**
24	Wing darkened apically. Legs yellow, only hind femur with preapical brown ring. Abdomen entirely black, basisternum without yellow spots. Body length 3.6. Thailand	***Strongylophthalmyia pectinigera* Shatalkin, 1996**
–	Wing with median cross-band and apical spot. Mid and hind femora with preapical brown ring. Hind tibia with subbasal brownish ring, occupying one third of tibia. Abdomen black, tergite 4 yellowish laterally, tergite 5 and 6 totally yellowish. Basisternum with a pair of yellow spots (Fig. 2e). Male unknown. Female body length 3.5–3.6. Vietnam (Figure 2)	***Strongylophthalmyia basisterna* sp. n.**
25	Face yellow. Frons entirely black. Palpus bicoloured: yellowish, darkened in basal half on anterior margin (Fig. 3c, d). Male unknown. Female body length 4.8. Vietnam (Figure 3)	***Strongylophthalmyia dichroa* sp. n.**
–	Face black. Palpus monochrom, entirely yellow or entirely black	**26**
26	Smaller: Male (female unknown) body length 2.3. Hind tibia yellow, brownish in middle. Male: first flagellomere with short conical process (Shatalkin 1996, Fig. 27). Thailand, Vietnam	***Strongylophthalmyia gibbifera* Shatalkin, 1993**
–	Larger: Body length 3.5–4.5. Hind tibia yellow or brownish basally. First flagellomere normal in male and female	**27**
27	Proximal section of M_1+2_ restricting discal cell before r-m approximately 0.5–0.6 times as short as distal section. Male palpus (Shatalkin 1996, Fig. 23) with one wide leaf-like scale apically. Body length 3.8. Burma	***Strongylophthalmyia freyi* Shatalkin, 1996**
–	Proximal section of M_1+2_ restricting discal cell before r-m approximately 0.7–0.8 times as short as distal section. Male palpus of different form.	**28**
28	Hind tibia slightly brownish in basal third. Male palpus yellow, with two or three black leaf-like scale apically (Shatalkin 1996, Fig. 22). Genitalia with aedeagal apodeme very long, more than two times as long as epandrium (Shatalkin 1996, Fig. 16). Body length 3.5–4.5. Taiwan; Burma, Vietnam, Japan, Russian Far East	***Strongylophthalmyia crinita* Hennig, 1940**
–	Hind tibia totally yellow. Male palpus dark, normal without black leaf-like scale. Genitalia with aedeagal apodeme very short and completely closed by epandrium (Shatalkin 1996, Fig. 21). Body length 3.5–3.7. Vietnam	***Strongylophthalmyia verrucifera* Shatalkin, 1996**
29	Wing smoky brown darkened. All coxae yellow. Body length 4.0. Indonesia (Java); Philippines, Thailand	***Strongylophthalmyia brunneipennis* (Meijere, 1914)**
–	Wing transparent, with or without median brownish cross-band on level of dm-cu and apical brownish spot	**30**
30	Wing clear, or brownish apically, without brownish cross-band on level of dm-cu	**31**
–	Wing with median cross-band and with apical spot	**32**
31	All coxae blackish. Face light brown to yellow. Frons with narrow yellow band in male and usually totally black in female. Fore femur black, yellowish apically; mid and hind femora totally black. Male first flagellomere without dorsal process. Male fore femur with large black setae on anterior surface and cercus extremely long, slender. Body length 2.3–2.6. Indonesia (Java, Sumatra), Thailand	***Strongylophthalmyia nigricoxa* (Meijere, 1914)**
–	All coxae yellow. Head black. Fore leg yellow, mid and hind femora blackish, yellow in basal third. Male first flagellomere with dorsal process long, S-shaped; arista long, as long as first flagellomere. Wing length 3.7; Head length (without antenna) 0.87. Taiwan, Thailand	***Strongylophthalmyia punctata* Hennig, 1940**
32	Fore tibia entirely blackish. Fore tarsus with segment 1 yellow basally and brown apically, segments 2–5 blackish brown. Body length 4.0. Indonesia (Sumatra), Thailand, Vietnam	***Strongylophthalmyia metatarsata* Meijere, 1919**
–	Fore tibia entirely or mostly yellow. Two or three basal segments of fore tarsus yellow	**33**
33	Vein r-m divides discal cell in half. Body length 4.0. Indonesia (Java, Sumatra), Thailand	***Strongylophthalmyia polita* Meijere, 1914**
–	Vein r-m divides discal cell in relation from 1:2.5 to 1:4.0	**34**
34	Mesonotum shining. Anepisternum covered with whitish setulae. Face yellowish. Palpus yellow. Mid tibia yellow in apical half. Body length 3.8–4.0. Philippines, Burma, Vietnam	***Strongylophthalmyia fascipennis* Frey, 1928**
–	Mesonotum matte. Anepisternum bare, with blackish short setae on posterior margin. Face dark brown, matte. Palpus black. Mid tibia entirely blackish	**35**
35	Fore femur black, narrowly yellow basally and apically, fore tibia yellow, brown in basal third; female with 3 distal segments of tarsi black (see also couplet 18) (Figure 7)	***Strongylophthalmyia stricta* sp. n.**
–	Fore femur yellow with black ring in apical third; fore tibia entirely yellow; female with 2 distal segments of tarsi black (see also couplet 18) (Figure 5)	***Strongylophthalmyia obtecta* sp. n.**

**Figure 3.**
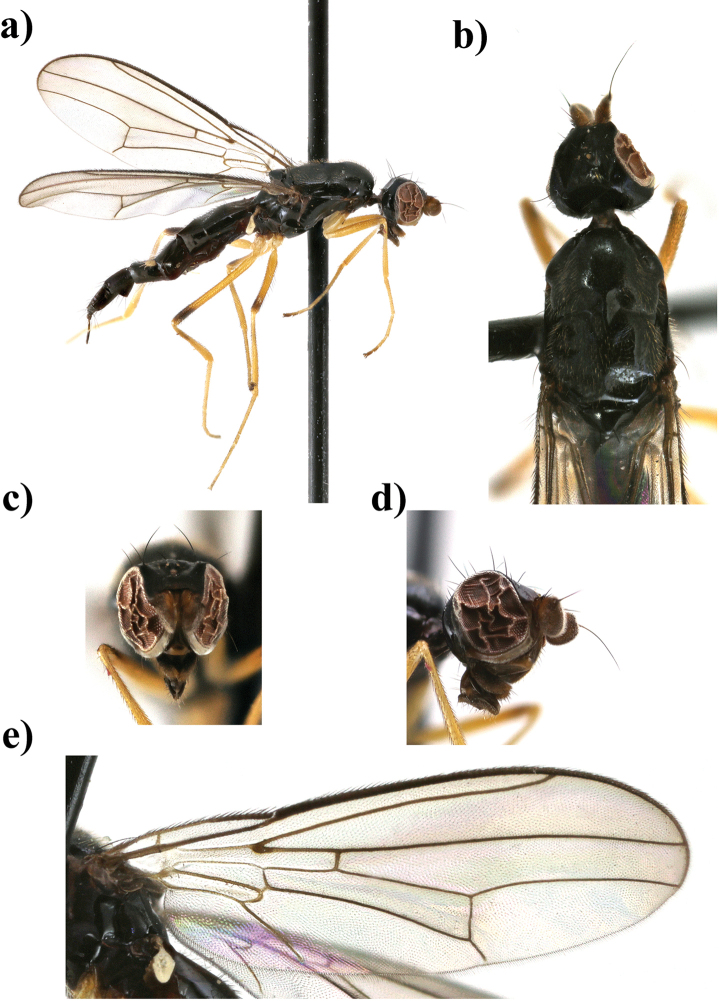
*Strongylophthalmyia dichroa* sp. n. **a** habitus, lateral view **b** head and thorax, dorsal view **c** head, anterior view **d** head, lateral view **e** basisternum **f** wing.

**Figure 4.**
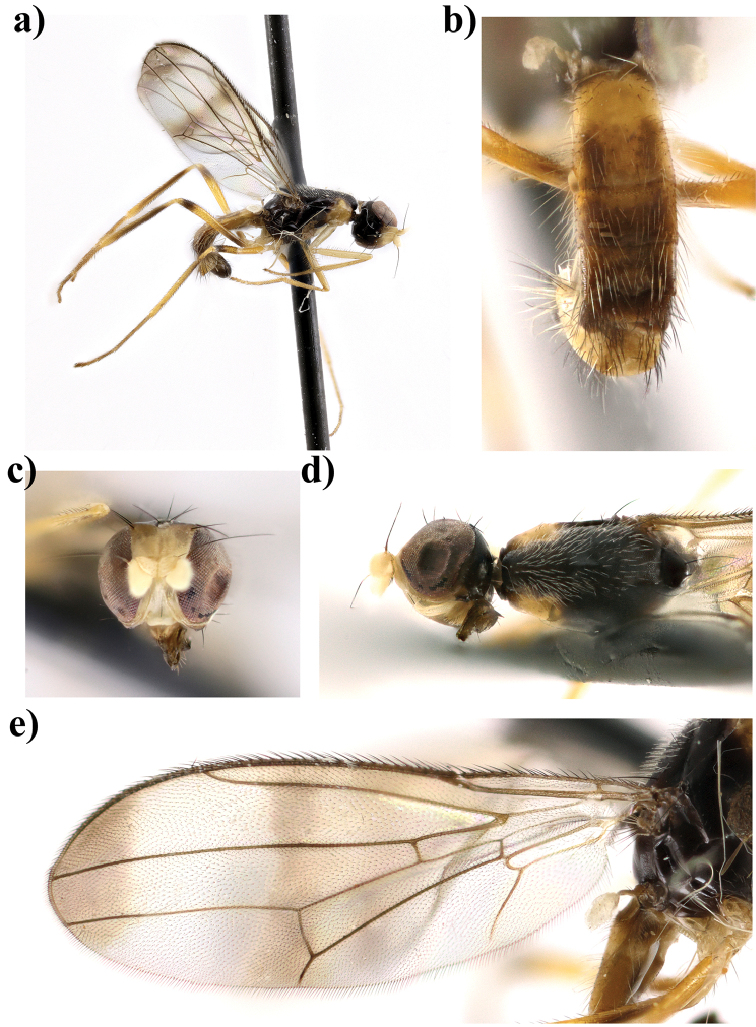
*Strongylophthalmyia gavryushini* sp. n. **a** habitus, lateral view **b** abdomen, dorsal view **c** head, anterior view **d** head and thorax, dorsal view **e** wing.

**Figure 5.**
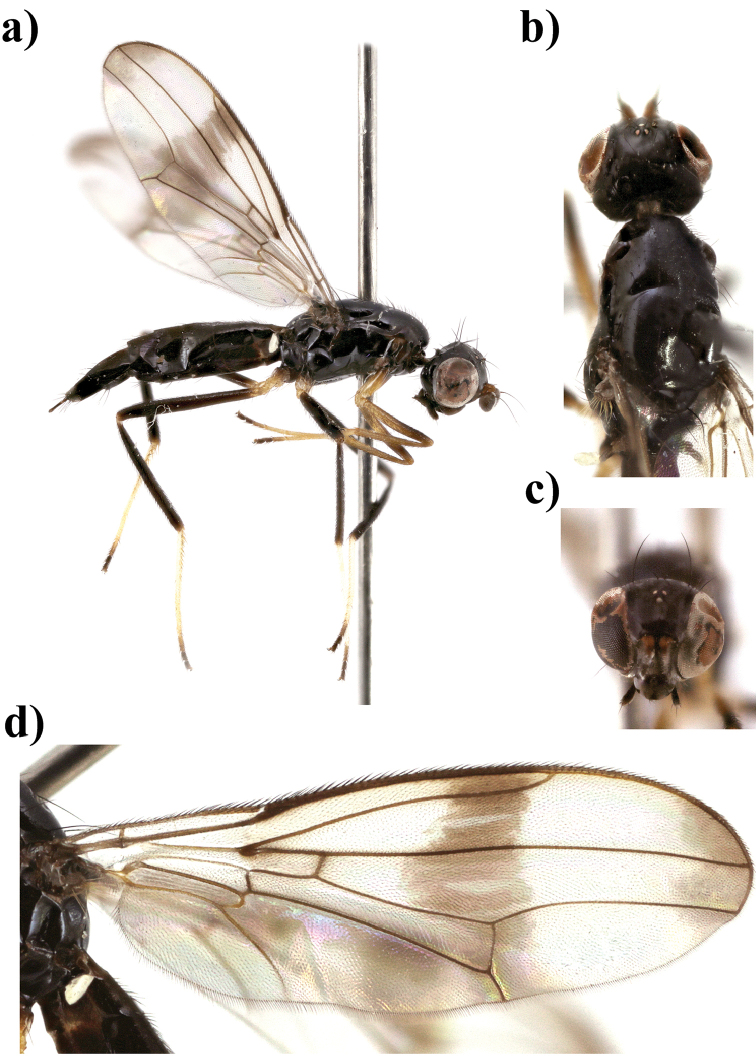
*Strongylophthalmyia obtecta* sp. n. **a** habitus, lateral view **b** head and thorax, dorsal view **c** head, anterior view **d** wing.

**Figure 6.**
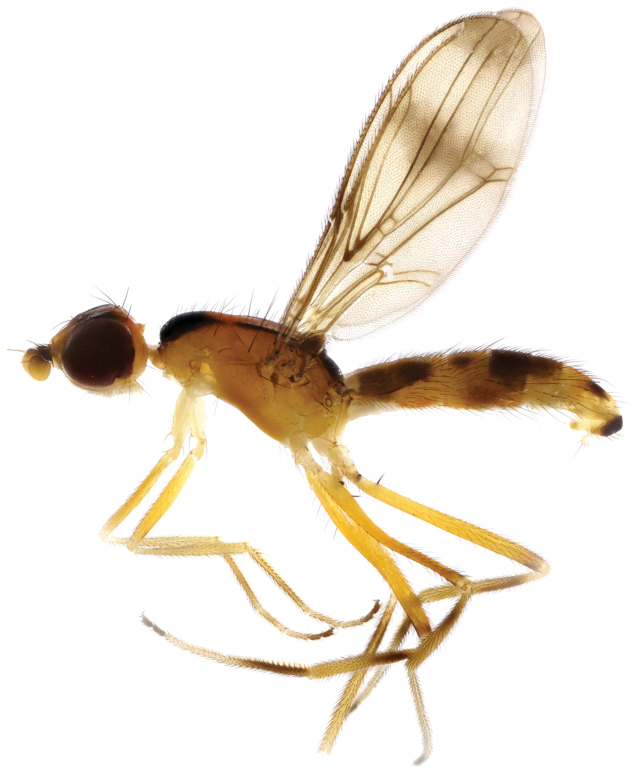
*Strongylophthalmyia orchidanthae* sp. n. Habitus, lateral view.

**Figure 7.**
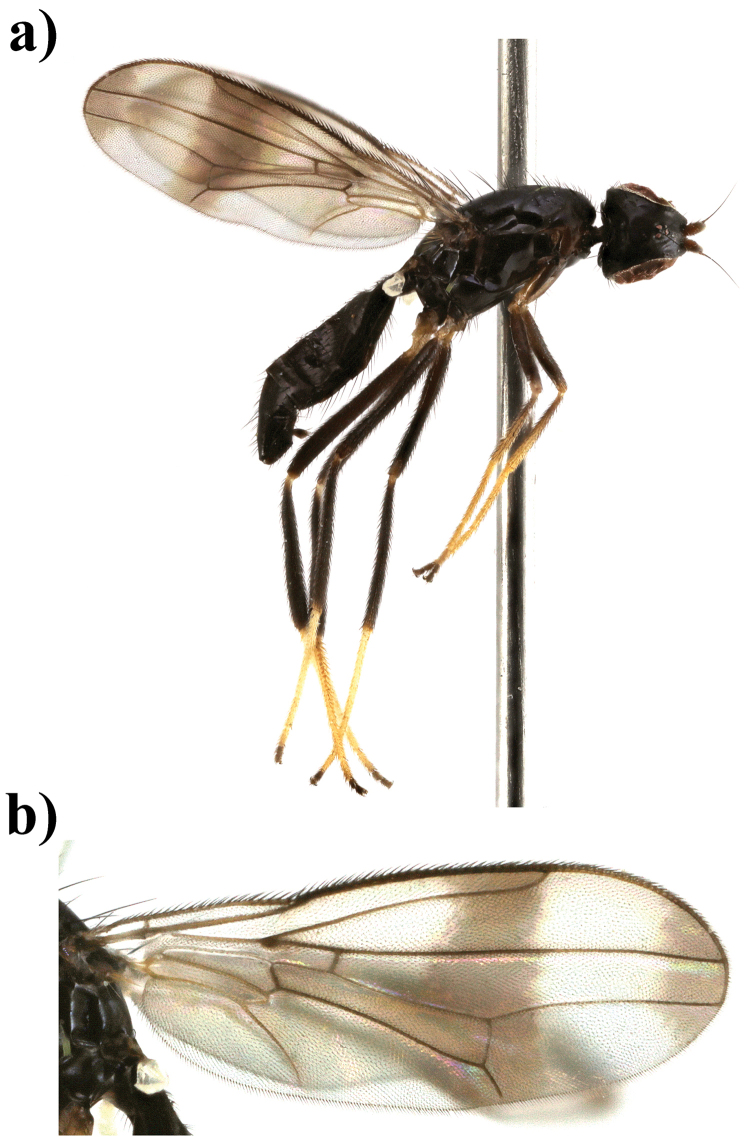
*Strongylophthalmyia stricta* sp. n. **a** habitus, lateral view **b** wing.

**Figure 8.**
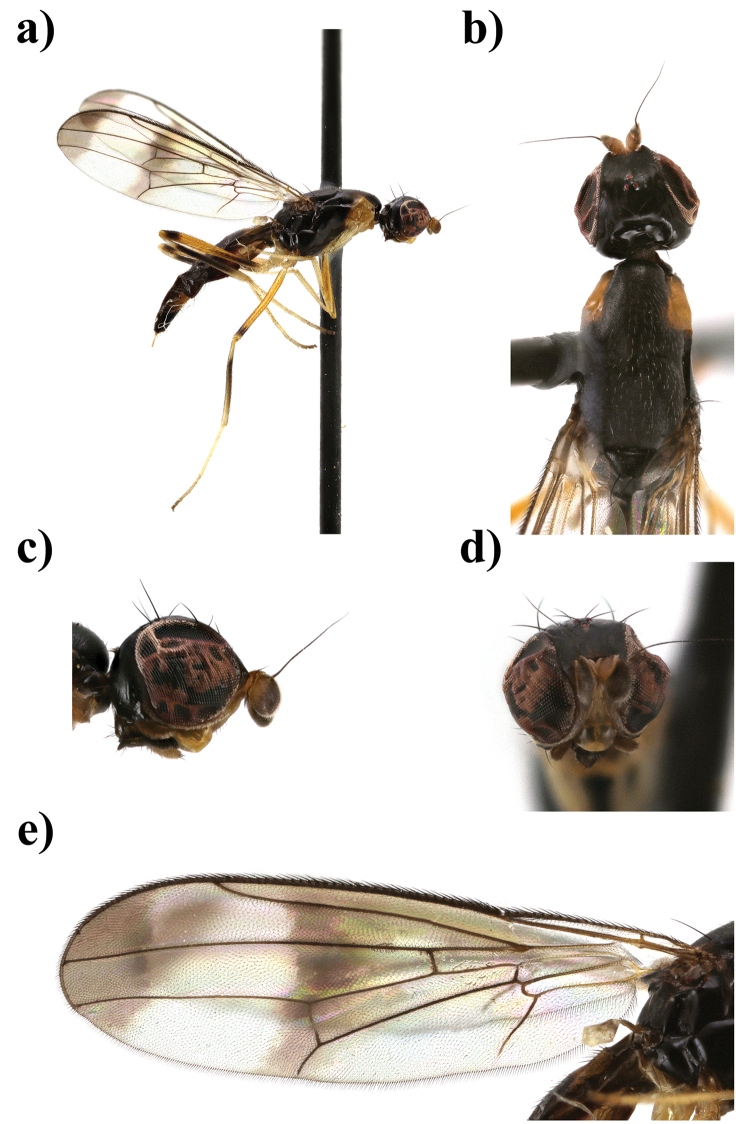
*Strongylophthalmyia tomentosa* sp. n. **a** habitus, lateral view **b** head and thorax, dorsal view **c** head, lateral view **d** head, anterior view **e** wing.

**Figure 9.**
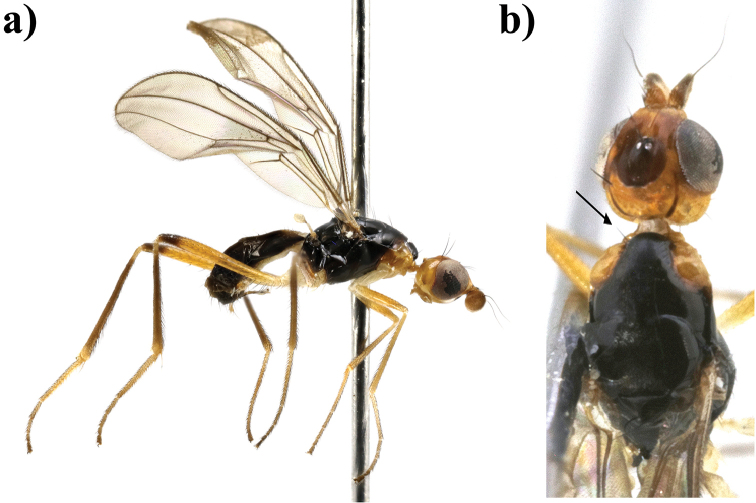
*Strongylophthalmyia splendida* Yang & Wang, 1996. **a** habitus, lateral view **b** head and thorax, dorsal view.

**Figure 10.**
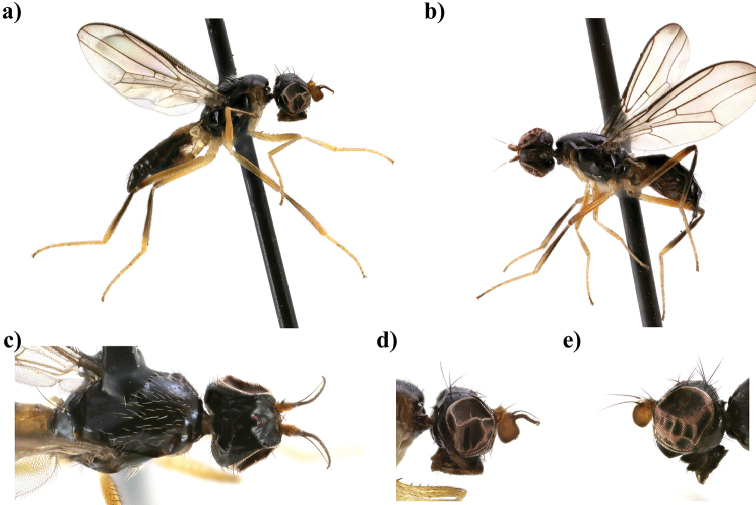
*Strongylophthalmyia thaii* L. Papp, 2006 **a** habitus male, lateral view **b** habitus female, lateral view **c** head and thorax, male, dorsal view; head, anterior view **d** head, male, lateral view **e** head, female, lateral view.

### Descriptions of new species

#### Strongylophthalmyia annulipes sp. n.

Taxon classificationAnimaliaDipteraStrongylophthalmyiidae

http://zoobank.org/6F1961E2-9838-4C98-A1BE-4B348AD527F5

Figure 1

##### Type material.

Holotype: 1 male, Vietnam, Lai Châu Province, Hoáng Liên (22.34997°N, 103.76818°E), 1947 m, 11.IV.2012 (D. Gavryushin). Paratypes: 2 female, Vietnam, Lai Châu Province, Hoáng Liên (22.347948°N, 103.769714°E), 1900 m, 16.IV and 19.IV.2012 (A.L. Ozerov); 1 male, 1 female, Vietnam, Lai Châu Province, Hoáng Liên (22.33788°N, 103.77922°E), 2068 m, 21.IV. and 7.V.2013 (T.V.Galinskaya). ZMUM.

##### Diagnosis.

This new species belongs to a group of species characterized by a yellow postpronotum, propleuron and basisternum. Three species from this group, *Strongylophthalmyia humeralis*, *Strongylophthalmyia bifasciata* and *Strongylophthalmyia yaoshana* are otherwise characterized by an arista with very short setulae and easily differentiated. Other species of this group, *Strongylophthalmyia spinosa*, *Strongylophthalmyia thaii*, *Strongylophthalmyia coarctata* Hendel, 1913 and *Strongylophthalmyia macrocera* differ from *Strongylophthalmyia annulipes* by transparent wings. *Strongylophthalmyia annulipes* sp. n. is close to *Strongylophthalmyia tomentosa* sp. n. and differs from it by the presence of a preapical black ring on the mid and hind femora and by black ring on the mid and hind tibia.

##### Description.

**Male.** *Head* black, shining, slightly longer than its height; frons entirely black, covered with grey tomentose of by very short setulae (0.01 mm). Occiput slightly convex (in dorsal view). Face black, matte, with row of short setulae along suture; parafacial black, covered with silvery grey tomentum, length of its setulae: 0.021–0.028 mm. Gena with brownish short stripe directly below parafacial. Antenna brownish yellow. First flagellomere rounded, its length almost equal to height; dark brown, narrowly yellow at base, with short yellow setulae dorsally. Arista dark brown, bare. Palpi brownish yellow. Chaetotaxy: three reclinate to lateroclinate orbital setae (the middle seta 2.5 times longer than others), 1 ocellar seta, 1 postocellar seta, 1 inner vertical seta, 1 outer vertical seta, 2 hair-like short frontal setae.

*Thorax* black. Postpronotum laterally, propleuron, basisternum, and anepisternum around spiracle yellow. Mesonotum matte, sparsely covered with short pale setulae; pleuron shiny; scutellum bare, matte. *Legs* yellow, mid and hind femora with dark brown preapical ring; mid tibia yellow basally and in apical half, and darkened between these yellow areas; hind tibia darkened, yellowish basally and in apical quarter. *Wings* with apical spot, with median transverse band on level of dm-cu vein and with weak darkening anteriorly at level of vein R_s_. R_2+3_ long: section of C between R_1_ and R_2+3_ 1.5 times longer than following section (between R_2+3_ and R_4+5_). R_4+5_ and M_1+2_ almost parallel apically. Section of M_1+2_ between r-m and dm-cu approximately 1.8 times longer than proximal section and 0.6 times shorter than distal section. Cell bm is 0.5 times shorter than discal cell. Calypter light grey with fan of very long light setulae on its margin. Halter with yellow stem and whitish knob. Chaetotaxy: one small postpronotal seta, one dorsocentral seta, two notopleural setae, one supraalar seta, one postalar seta, one anepisternal seta, one stout apical scutellar seta. All setae black.

*Abdomen* shiny black, narrowly yellow basally.

Body length 4.2 mm. Wing length 3.8 mm.

**Female** differs from male in following characters. Frontal setulae very short, hardly visible. Palpi dark brown. Mesonotum covered with short setulae. Mid tibia widely darkened, yellowish basally and in apical quarter. Body length 3.8–4.7 mm; wing length 3.5–4.2 mm.

##### Etymology.

The specific name refers to black ring on mid and hind femora.

#### Strongylophthalmyia basisterna sp. n.

Taxon classificationAnimaliaDipteraStrongylophthalmyiidae

http://zoobank.org/8A8E266B-04BC-4B10-A229-5C37EBFC6ECF

Figure 2

##### Type material.

Holotype: 1 female, Vietnam, Lai Châu Province, Hoáng Liên (22.347948°N, 103.769714°E), 1700 m, 22.V.2014 (A.L. Ozerov). Paratype: 1 female, Vietnam, Lai Châu Province, Hoáng Liên (22.347948°N, 103.769714°E), 1900 m, 22.V.2014 (A.L. Ozerov); 1 female, Vietnam, Lai Châu Province, Sa Pa env. (22.3872°N, 103.7867°E), 1682 m 23.V.2014 (D. Gavryushin) ZMUM.

##### Diagnosis.

This new species strongly differs from all species from Vietnam and neighbour countries. It is morphologically close to *Strongylophthalmyia puncticollis* Frey, 1928 (from Philippines and Papua New Guinea) and to *Strongylophthalmyia fasciolata* Meijere, 1919 (from Sumatra). *Strongylophthalmyia puncticollis* differs from the new species by having the abdomen entirely black; all femora have a brown preapical ring, the hind tibia is black basally and apically and yellow in the median third. *Strongylophthalmyia fasciolata* differs from the new species by an entirely black abdomen, black matte mesonotum, dark legs, and halteres with a brownish stem. In the key by Steyskal (1971) *Strongylophthalmyia fasciolata* is close to *Strongylophthalmyia angusticollis* Frey, 1956 (from Burma). The last species is characterized by the arista covered with short setulae.

##### Description.

**Female.** *Head* black, 1.3 times longer than height. Frons matte with yellowish brown spot between antenna and eye. Ocellar tubercle slightly shifted anteriorly: the ratio between height of the frons from its anterior margin to hind ocelli and from the hind ocelli to the vertex or vti equal to 1.3. Occiput poorly convex (in dorsal view). Face dark brown, matte, with row of short setulae along suture. Parafacial yellow, covered with whitish tomentum, setulae on parafacial around 0.5 times shorter than setulae along facial suture. Antenna dark brown. First flagellomere 1.1 times longer than high, dark brown with long yellowish marginal setulae. Arista dark brown, bare. Mouthparts dark, palpi darkish brown. Chaetotaxy: two reclinate to lateroclinate orbital setae (posterior seta 2.0 times longer than anterior), 1 oc, 1 poc, 1 vti, 1 vte, frontal setae absent.

*Thorax* black, shining. Basisternum with two bright yellow spots between fore coxae; yellowish brown stripe extended from postpronotum over anterior spiracle to coxa. Mesonotum shining, with 4 rows of short yellow setae along dc and ac rows. Scutellum matte. *Legs* yellow, mid and hind femur with preapical brown ring (this ring narrower on mid femur); mid and hind tibiae with subbasal brownish ring; two last tarsal segments black. *Wings* with apical spot, median transverse band through dm-cu and with weak darkening in anterior part of R_s_. Right border of median band situated slightly laterally to R_2+3_ vein. R_2+3_ long: section of C between R_1_ and R_2+3_ 1.3 times longer than following section (between R_2+3_ and R_4+5_). R_4+5_ and M_1+2_ almost parallel apically. Section of M_1+2_ between r-m and dm-cu slightly concave, 3 times longer than proximal section and 0.9 times shorter than distal section. Cell bm 0.5 times shorter than discal cell. Calypter brownish yellow with fan of very long yellowish setulae on its margin. Halter with yellow stem and whitish knob. Chaetotaxy: one small pprn, one pprn, one dc, ac in two rows, two npl, one sa, one pa, one anepst, scutellum with a pair of stout setae apically. All setae black.

*Abdomen* black, shiny; tergite 4 laterally, tergite 5, 6 totally yellow.

Body length 5.6 mm (5.5 and 6.5 in paratypes); wing length 5.2 mm (5.2 and 5.3 in paratypes).

**Male** unknown.

##### Etymology.

The specific name refers to the two bright yellow spots on basisternum.

#### Strongylophthalmyia dichroa sp. n.

Taxon classificationAnimaliaDipteraStrongylophthalmyiidae

http://zoobank.org/41F487F6-4F57-4BE6-B9AE-506B4A0DFBA0

Figure 3

##### Type material.

Holotype: 1 female, Vietnam, Lai Châu Province, Hoáng Liên (22.33788°N, 103.77922°E), 2068 m, 7.V. 2013 (T.V. Galinskaya). ZMUM.

##### Diagnosis.

This new species belongs to the *Strongylophthalmyia crinita* species group. Species of this group are characterized by transparent wing, bare arista, yellow legs, mid and hind femora with apical dark brown ring. Within this group the new species is close to species with yellow face and totally black frons. The new species differ from all species of *Strongylophthalmyia crinita* species group by palpus with character coloration (yellow, darkened in basal half on anterior margin, with some black setulae apically). Among oriental species only *Strongylophthalmyia stylocera* from Philippines has these characters, but it is a much smaller species, 2.7 mm (4.8 mm in *Strongylophthalmyia dichroa*).

##### Description.

**Female.** *Head* black, shining, slightly shorter than height. Frons entirely black, shining; ocellar tubercle slightly shifted towards anterior: the ratio between height of the frons from its anterior margin to hind ocelli and from hind ocelli to vertex or inner vertical seta equal to 1.1. Occiput black shining, slightly convex. Face very narrow, yellowish, with triangular black spot in lower part, and consequently it seems dark; face with row of short setulae along suture. Parafacial yellow, covered with white tomentum. Basal antennal segments yellow, first flagellomere darkened, rounded, with short yellow dorsal setulae. Arista brown, bare. Mouthparts brown; palpus yellow, darkened in basal half on anterior margin, with some black setulae apically. Chaetotaxy. Three orbital setae, 1 ocellar seta, 1 postocellar seta, 1 inner vertical seta, 1 outer vertical seta, 1 frontal seta.

*Thorax* black. Basisternum without a pair of yellow spots. Mesonotum slightly matte, clothed with short yellow dense setulae; postpronotum shining, practically bare; pleuron, including region surrounding anterior spiracle black shining, anepisternum with well-developed light or yellowish setulae ventrally near the mid-coxa and posteriorly near the pleural suture; scutellum bare, matte. *Legs* yellow, mid and hind femora with dark brown preapical ring; hind tibia with traces of darkening in the basal half; last two tarsal segments slightly brownish. *Wings* transparent; cell r_4+5_ and posterior border of cell r_2+3_ slightly smoke-coloured. Vein R_2+3_ long, its end far beyond the level of dm-cu: section of C between R_1_ and R_2+3_ in 1.9 times longer than a projection of a following section (between R_2+3_ and R_4+5_). R_4+5_ and M_1+2_ nearly parallel apically. Vein M_1+2_ between r-m and dm-cu approximately 2 times longer than previous one and nearly 1.4 times shorter than ultimate one. Cell bm approximately 0.45 times shorter than discal cell. Calypter dark grey with fan of very long light setulae on margin. Halter with yellowish stem and whitish knob. Chaetotaxy: one very small postpronotal seta, one dorsocentral seta, two notopleural setae, one supraalar seta, one postalar seta, one anepisternal seta, one stout apical scutellar seta. All setae black.

*Abdomen* shiny black.

Body length 4.8 mm; wing length 4.0 mm.

**Male** unknown.

##### Etymology.

The specific name refers to a bi-coloured palpus.

#### Strongylophthalmyia gavryushini sp. n.

Taxon classificationAnimaliaDipteraStrongylophthalmyiidae

http://zoobank.org/B5F8CFA3-5B49-4068-A239-C8FD3D689163

Figure 4

##### Type material.

Holotype: 1 male, Vietnam, Lai Châu Province, Sa Pa env. (22.3872°N, 103.7867°E), 1682 m, 25.V.2014 (D. Gavryushin) ZMUM

##### Diagnosis.

Species of *Strongylophthalmyia* are characterized by a slight shift of the ocellar tubercle anteriorly and in some species, the ocellar tubercle can be situated in the middle of the frons. *Strongylophthalmyia gavryushini* sp. n., however, has the ocellar tubercle positioned nearly on the edge of the vertex. The value of frontal index (the ratio between height of the frons from its anterior margin to hind ocelli and from hind ocelli to vertex or vti) is high, equal to 4.3. The frontal index of the other new species of *Strongylophthalmyia* described in this paper vary from 1 up to 1.7. Only *Strongylophthalmyia tomentosa* sp. n. has the frontal index equal to 2.7.

The new species is characterised by fore femur with row of ten black setae dorsally and with row of long dense yellowish setulae bent lateroapically aside of femoral apex, proximally these setulae 1.3 longer than distally. Tergite six with long black setulae dorsally and 6–7 long yellowish setulae laterally (Figure 4b). The new species is close to *Strongylophthalmyia trifasciata* Hennig. Males of these species are similar by abdomen partially yellow. *Strongylophthalmyia trifasciata* differs by wing with a short cross-band in anterior part at a level of R_s_. At least last abdominal tergites of male are yellow, with black transverse band, tergite six with patch of thick black setae laterally (fig. 20 in Hennig 1941).

##### Description.

**Male.** *Head* yellow, length almost equal to its height. Frons yellow, black shining posteriorly to hind margin of ocellar tubercle. Frontal index 4.3. Occiput black, slightly convex. Gena yellow. Face yellow, with row of short setulae along suture. Parafacial covered with silvery grey tomentum. Antenna light yellow. First flagellomere 1.6 times longer than width, with short yellow dorsal marginal setulae. Arista dark brown, basally yellow, bare. Palpus yellow. Clypeus dark brown. Frons between upper orbital and outer vertical setae with one short setula. Chaetotaxy: Two orbital setae (anterior – 0.18 mm, posterior – 0.24 mm); 1 ocellar seta, 1 postocellar seta, 1 inner vertical seta, 1 outer vertical seta, 1 hair-like very short frontal seta.

*Thorax* black. Postpronotum laterally and dorsally, propleuron, anepisternum around spiracle yellow. Mesonotum matte, sparsely covered with short yellow setulae; pleuron shiny; scutellum bare, matte. *Legs* yellow, mid and hind femora with dark brown preapical ring, hind femur brownish basally; mid tibia with brownish spot in basal quarter; hind tibia brown, yellow basally and apically. Fore femur with prominent row of ten black setae dorsally and with row of long dense yellowish setulae bent lateroapically aside of femoral apex, proximally these setulae almost as long as femor width. Mid tibia with two spurs, yellow and black, both 0.1 mm. *Wings* with apical spot, median transverse band at level of dm-cu and with weak darkening in anterior part at level of R_s_. Vein R_2+3_ long, merged with C vein far from the level of dm-cu: section of C between R_1_ and R_2+3_ 1.6 times longer than section between R_2+3_ and R_4+5_. Veins R_4+5_ and M_1+2_ almost parallel apically. Section of M_1+2_ between r-m and dm-cu nearly 1.5 times longer than proximal section and around 0.6 times shorter than distal section. Cell bm approximately 0.4 times shorter than discal cell. Calypter brownish grey with fan of very long dark setulae on margin. Halter with yellow stem and whitish knob. Chaetotaxy: two small postpronotal seta, one dorsocentral seta, two notopleural setae, one supraalar seta, one postalar seta, one anepisternal seta, one stout apical scutellar seta (0.42 mm). All setae black.

*Abdomen* yellow, matte, with light brownish spots and strips (Figure 4b). Tergites with long dark brownish setulae, length of these setulae increased posteriorly; tergite six with 6–7 long yellowish setulae laterally.

Body length 3.4 mm; wing length 3.2 mm.

**Female** unknown

##### Etymology.

The species is named after our colleague Dr. D.I. Gavryushin.

#### Strongylophthalmyia obtecta sp. n.

Taxon classificationAnimaliaDipteraStrongylophthalmyiidae

http://zoobank.org/6BB4B76A-CC6A-4FF0-A283-3F9382A3F6F5

Figure 5

##### Type material.

Holotype: 1 female, Vietnam, Lai Châu Province, Hoáng Liên (22.347948°N, 103.769714°E), 1900 m, 18.IV.2012 (A.L. Ozerov). Paratype: 1 female, Vietnam, Lai Châu Province, (22.347948°N, 103.769714°E), 1947 m, 22.V.2014 (D. Gavryushin) ZMUM.

##### Diagnosis.

*Strongylophthalmyia obtecta* sp. n. and *Strongylophthalmyia stricta* sp. n. have the thorax with 2 dc and two rows of black setulae on the line of dc with one large setula before transverse suture, 2 rows of long black ac. Based on these characters both species are close to *Strongylophthalmyia raricornis* Shatalkin, 1981; *Strongylophthalmyia raricornis* differs from two new species by first flagellomere bilobate. *Strongylophthalmyia obtecta* sp. n. differs from *Strongylophthalmyia stricta* sp. n. in having fore femur and tibia yellow; and by having the two distal segments of the tarsus black (distal three segments black in female of *Strongylophthalmyia stricta* sp. n.)

##### Description.

**Female.** *Head* entirely black, 0.9 times shorter than height; frons shining, entirely black, upper occiput slightly convex. Gena narrow; postgena broad, approximately 0.5 times shorter than eye height. Face dark brown, matte, with row of short setulae along suture. Parafacial with silvery grey tomentum, these setulae 0.25 times shorter than setulae along facial suture. Scape and pedicel yellowish brown, first flagellomere length almost equal to height. First flagellomere dark brown, with long pale marginal setulae, nearly 3 times less than flagellomere width. Arista dark brown, bare. Mouthparts and palpus black. Chaetotaxy: three orbital setae (medial seta 1.8 times longer than others); 1 ocellar seta, 1 postocellar seta, 1 inner vertical seta, 1 outer vertical seta; hair-like short frontal setae present.

*Thorax* black. Postpronotum laterally, proepisternum, anepisternum behind anterior spiracle yellowish brown, shining. Mesonotum matte, with rows of black setulae; dc row of setae includes 1+2 large dc; pleuron shining; scutellum slightly shiny. *Legs*. Fore coxa yellow with blackish stripe on anterior surface basally, mid and hind coxae yellow. Fore femur yellow, with blackish ring in apical third; mid and hind femora black, narrowly yellowish basally; fore tibia yellow, mid and hind tibia black, tarsi yellow, segment 3 brown, segments 4–5 dark brown to black. *Wings* with apical spot, median transverse band on level of dm-cu and with light brown spot anteriorly on level of R_s_. Distal border of median band nearly reaching apex of vein R_2+3_. Vein R_2+3_ long: section of C between R_1_ and R_2+3_ 1.5 times longer than section between R_2+3_ and R_4+5_. Veins R_4+5_ and M_1+2_ almost parallel apically. Section of M_1+2_ between r-m and dm-cu slightly concave, 2.5 times longer than proximal section and 0.7 times shorter than distal section. Cell bm 0.4 times shorter than discal cell. Calypter brownish grey with fan of very long yellowish setulae on margin. Halter with brownish stem and whitish knob. Chaetotaxy: one short postpronotal seta, two dorsocentral seta and two rows of black setulae on the line of dc with one large setula before transverse suture, two rows of long black acrostichal setulae, two notopleural setae, one supraalar seta, one postalar seta, one anepisternal seta, one stout apical scutellar seta and one short discal scutellar seta in front of apical ones. All setae black.

*Abdomen* shiny black, with brownish tinge on anterior margin of tergite 1.

Body length 4.2 mm (4.6 in paratype); wing length 3.7 mm (4.1 in paratype).

**Male** unknown.

##### Etymology.

Obtectus (Latin) = matted. In this case it refers to the setulae covering the mesonotum.

#### Strongylophthalmyia orchidanthae sp. n.

Taxon classificationAnimaliaDipteraStrongylophthalmyiidae

http://zoobank.org/DDCAAC2F-4088-4CAF-A04E-C3F2FF18D565

Figure 6

##### Type material.

Holotype: 1 male, Vietnam, Phu Tho province, Thanh Son district, Xuan Son National Park, (21°6'45"N, 104°57'25"E.), 23.X.2014 (T.V. Galinskaya). ZMUM.

##### Diagnosis.

*Strongylophthalmyia orchidanthae* sp. n. is superficially similar to the *Strongylophthalmyia lutea* species group in having a yellow thorax. *Strongylophthalmyia lutea* species group is characterized by vein R_2+3_ short and section of C between R_1_ and R_2+3_ 0.7 times shorter than distal section between R_2+3_ and R_4+5_. The new species has section of C between R_1_ and R_2+3_ approximately equal to distal section. This new species is similar to *Strongylophthalmyia immaculata* Hennig, 1940 from Formosa, which is included in the *Strongylophthalmyia lutea* group, i.e. characterized by short vein R_2+3_. but *Strongylophthalmyia immaculata* differs from *Strongylophthalmyia orchidanthae* by armed fore femur, abdomen entirely black, mesonotum without pair of brown longitudinal stripes, wing transparent. *Strongylophthalmyia nigriventris* Frey, 1928 from Philippines, Malaysia and Papua New Guinea is characterized by a pair of brown stripes coalesced in anterior part of mesonotum, as in this new species. It differs from *Strongylophthalmyia orchidanthae* sp. n. by abdomen entirely black, legs entirely yellow and pedicel yellow, first flagellomere brownish.

##### Description.

**Male.** *Head* yellow, 1.3 times longer than height, ocellar tubercle small, black; frons slightly widened towards vertex, frontal index equal to 1.3. Occiput slightly convex. Face yellow with row of yellow setulae along suture. Parafacial covered with silvery grey tomentum. Antenna yellow, pedicel black. First flagellomere slightly brownish basally, its length around 0.9 times shorter than high. Arista dark yellow, bare. Mouthparts and palpus yellow. Chaetotaxy: three orbital setae, 1 ocellar seta, 1 postocellar seta, 1 inner vertical seta, 1 outer vertical seta.

Thorax yellow with anterior 3/4 of presutural scutum black with one pair of black lines continuing along dorsocentral rows onto sides of scutellum. A pair of postsutural longitudinal stripes not merged with anterior arcuate area; in posterior part of mesonotum these stripes continued on scutellum. Scutellum yellow with black border laterally. Mediotergite blackish brown. *Legs* yellow, with coxae and tarsi white, two distal tarsal segments brown. Mid femur with light brownish preapical ring, mid tibia with brown subbasal band. Hind femur with light brown subapical ring; hind tibia with brown subbasal band; basal segment of hind tarsus darkened. *Wings* with brown apical spot and cross-band at level of vein dm-cu. Vein R_2+3_ long: section of C between R_1_ and R_2+3_ nearly 1.1 times longer than section between R_2+3_ and R_4+5_. Section of M_1+2_ between r-m and dm-cu nearly 2.3 times longer than proximal section and 0.7 times shorter than distal section. Posterior basal cell approximately 0.6 times shorter than discal cell. Calypter yellowish with fan of very long yellow setulae on margin. Halter with yellow stem and whitish knob. Chaetotaxy: one very small postpronotal seta, one dorsocentral seta, two notopleural setae, one supraalar seta, one postalar seta, one anepisternal seta, one stout apical scutellar seta.

*Abdomen* yellow, tergite 1–2 with pair of black longitudinal stripes, tergite 3 with pair of light brown median spots, tergite 4 with large black triangular spot, tergite 5 with narrow dorsal band on anterior ¾. Sternite 8 large, situated on dorsal side, with large black spot. Epandrium yellow apically, brown basally.

Body length 4.3 mm; wing length 3.1 mm.

**Female** unknown

##### Etymology.

The new species was collected on *Orchidantha* (Zingiberaceae).

#### Strongylophthalmyia stricta sp. n.

Taxon classificationAnimaliaDipteraStrongylophthalmyiidae

http://zoobank.org/6EDEDB8E-FE8D-4F51-AA68-BB1396B66110

Figure 7

##### Type material.

Holotype: 1 male, Vietnam, Lai Châu Province, Sa Pa env. (22.330396°N, 103.82418°E), 1284 m, 12.IV.2012 (A.L. Ozerov) ZMUM. Paratypes: 1 male, Vietnam, Lai Châu Province, Sa Pa env. (22.1454°N, 103.8053°E), 1448 m 21.V.2014 (D. Gavryushin); 1 female, Vietnam, Lai Châu Province, Hoáng Liên (22.347948°N, 103.769714°E), 1900 m, 22.V.2014 (A.L. Ozerov).

##### Diagnosis.

*Strongylophthalmyia stricta* sp. n. and *Strongylophthalmyia obtecta* sp. n. have the thorax with 2 dc and two rows of black setulae on the line of dc with one large setula before transverse suture, 2 rows of long black ac. Based on these characters both species are close to *Strongylophthalmyia raricornis* Shatalkin, 1981; *Strongylophthalmyia raricornis* differs from two new species by first flagellomere bilobate. *Strongylophthalmyia obtecta* sp. n. differs from *Strongylophthalmyia stricta* sp. n. by fore femur and tibia yellow; two distal segments of tarsi black (instead of three distal segments in female of *Strongylophthalmyia stricta* sp. n.).

##### Description.

*Head* entirely black, its length equal to its height. Frons shining, entirely black. Occiput slightly convex. Face brownish, with row of short setulae along suture. Parafacial covered with short silvery grey tomentum. Antenna yellowish brown, first flagellomere 1.5 times longer than high. First flagellomere yellow with dorsum dark brown, with long pale marginal setulae. Arista dark brown, bare. Mouthparts and palpus black. Chaetotaxy: Three orbital setae, 1 ocellar seta, 1 postocellar seta, 1 inner vertical seta, 1 outer vertical seta, 3 hair-like short frontal setae.

*Thorax* black. Postpronotum laterally, proepisternum, anepisternum behind anterior spiracle yellowish brown, shining. Mesonotum matte, with rows of black setulae; 1+3 large dorsocentral setae; pleuron shining; scutellum matte. *Legs*. Fore coxa black with anterolateral margin of fore coxa yellowish and mid and hind coxae yellowish distally, fore femur black, narrowly yellowish basally and apically; mid and hind femora black; fore tibia yellow with brownish ring in basal third; mid and hind tibia black, tarsi yellow, two distal segments black. Hind trochanter with a posterior round blackish spot; hind femur basally with round posteroventral process, without 3–4 ventral setulae (as in male of *Strongylophthalmyia papuana*), and with small subbasal black posterior process with two yellow setulae on it and with two yellow setae on trochanter distally to this process. *Wings* with apical spot, median transverse band at level of dm-cu and light brown spot anteriorly at level of R_s_. Distal border of median band reaching apex of vein R_2+3_. Vein R_2+3_ long: section of C between R_1_ and R_2+3_ 1.3 times longer than penultimate section (between R_2+3_ and R_4+5_). Veins R_4+5_ and M_1+2_ almost parallel apically. Proximal section of M_1+2_ between r-m and dm-cu 2.7 times longer than proximal one and 0.7 times shorter than distal one. Cell bm 0.4 times shorter than discal cell. Calypter brownish grey, with fan of very long light setulae on margin. Halter with brownish stem and whitish knob. Chaetotaxy: one small postpronotal seta, 1+3 dorsocentral setae, two rows of acrostichal setulae, two notopleural setae, one supraalar seta, one postalar seta, one anepisternal seta, one stout apical scutellar seta and one short discal scutellar seta in front of apical seta. All setae black.

*Abdomen* black, shining, with two yellowish spots dorsally on first tergite.

Body length 3.3 mm; wing length 2.9 mm.

**Female** differs by having three black distal tarsal segments; abdomen totally black; trochanter without processes.

Body length 4.8 mm; wing length 4.2 mm.

##### Etymology.

*Strongylophthalmyia stricta* is characterized by a dense field of adjoining setulae on the scutum that are sticking out or protrusive (*strictus* in Latin), and rarely ordered in regular lines.

#### Strongylophthalmyia tomentosa sp. n.

Taxon classificationAnimaliaDipteraStrongylophthalmyiidae

http://zoobank.org/D592F9BC-9BC5-42EE-AF71-E4F9960805DA

Figure 8

##### Type material.

Holotype: 1 female, Vietnam, Lai Châu Province, Tam Duong Distr. (22.37017°N, 103.75793°E), 1745 m, 26.X.2015 (D.Gavryushin). Paratype: 1 female, Vietnam, Lai Châu Province, Hoáng Liên (22.347948°N, 103.769714°E), 1900 m, 11.IV.2012 (A.L.Ozerov).

##### Diagnosis.

The new species belongs to a large and varied group of species with a yellow postpronotum. Within this group, *Strongylophthalmyia tomentosa* sp. n. is close to *Strongylophthalmyia bifasciata* Wang & Yang from China (Zhejiang) on the basis of a longitudinal band running along R2+3 from its base to the medial transverse band. *Strongylophthalmyia dorsocentralis* has a similar wing band, but this species differs from first two species by many characters, including the coloration of the thorax and by the absence of microsetulae across the mesonotum. *Strongylophthalmyia tomentosa* sp. n. differs from *Strongylophthalmyia bifasciata* by the shorter vein R_2+3_, smaller size of apical spot and by bare arista. *Strongylophthalmyia tomentosa* sp. n. is also similar to *Strongylophthalmyia annulipes* sp. n. (see the diagnosis of *Strongylophthalmyia annulipes*).

##### Description.

**Female.** *Head* slightly shorter than height. Frons matte black, narrowly yellow anteriorly. The ratio between height of the frons from its anterior margin to hind ocelli to the distance from the hind ocelli to vertex or vti is equal to 2.7. Occiput black, shining, slightly convex. Face yellow with row of short setulae along suture, clypeus yellow shining; parafacial yellow with silver grey tomentum. Gena black with small brownish spot between lateral margin of mouth and parafacial. Antenna yellow, first flagellomere rounded, 0.9 times shorter than height, dark brown on inner surface, narrowly yellow basally, widely dark brown apically and on dorsal margin externally, with short yellow dorsal setulae. Arista dark brown, bare. Mouthparts dark brown; palpus yellow, with short and thick black setulae apically. Chaetotaxy: Two orbital setae (posterior seta 2.6 times longer than anterior seta), 1 ocellar seta, 1 postocellar seta, 1 inner vertical seta, 1 outer vertical seta, frontal setae absent.

*Thorax* black. Postpronotum, proepisternum, basisternum, anepisternum around of spiracle yellow, and yellow coloration on postpronotum slightly extending to mesonotum (Figure 8a,b). Mesonotum matte, sparsely covered with short pale setulae; pleuron shining; scutellum bare, matte. *Legs*. Fore legs yellow, fore femur externally with pale brownish spot basally and apically. Mid legs yellow, mid femur with dark brown preapical ring, mid tibia: yellow, dark brown medially. Hind legs yellow, hind femora with dark brown preapical ring and with brown spot basally, hind tibia blackish, yellow basally and in apical quarter; fore tarsi yellow, mid tarsi yellow with brownish apical segment, hind tarsi yellow with three apical segments brownish. *Wings* with strong apical band, median transverse band on level of dm-cu (section of this vein between C and R_2+3_ undeveloped). R_2+3_ long: section of C between R_1_ and R_2+3_ 1.5 times longer than distal section (between R_2+3_ and R_4+5_). R_4+5_ and M_1+2_ almost parallel apically. Section of M_1+2_ between r-m and dm-cu 1.8 times longer than proximal section and 0.6 times shorter than distal section. Cell bm 0.6 times shorter than discal cell. Calypter light grey with fan of very long light setulae on margin. Halter with yellow stem and whitish knob. Chaetotaxy: one very small postpronotal seta, one dorsocentral seta, two notopleural setae, one supraalar seta, one postalar seta, one anepisternal seta, one stout apical scutellar seta. All setae black.

*Abdomen* black, shining, tergite 1 and basal half of tergite 2 yellow, and one pair of lateral yellow spots on anterior margin of tergite 6 extending to two thirds of its length.

Body length 3.6 mm; wing length 3.7 mm.

**Male** unknown.

##### Etymology.

The mesonotum of the new species is covered with short setulae, giving it the appaerance of a felt surface.

### Species new to the fauna of Vietnam

#### Strongylophthalmyia angusticollis

Taxon classificationAnimaliaDipteraStrongylophthalmyiidae

Frey

Strongylophthalmyia angusticollis Frey, 1956: 132 (male, female)

##### Material.

1 male, Vietnam, Lai Châu Province, Hoáng Liên (22.347948°N, 103.769714°E), 1900 m, 16.IV.2012 (A.L. Ozerov); 1 female, same locality, 18.IV.2012 (A.L. Ozerov). ZMUM.

##### Diagnosis.

*Strongylophthalmyia angusticollis* is characterized by the following combination of characters. Frons black, shining, yellow in anterior one third or quarter. Face yellow. Arista dark brown, pubescent. Thorax black, basisternum yellow in male or with a pair of yellow spots in female (original description lack these characters). Mesonotum matte, sparsely covered with short yellowish setulae. 1 dc. Legs yellow, mid and hind femora with preapical brown ring; mid and hind tibiae with wide subbasal brownish ring. Wings with apical spot, median transverse band at level of dm-cu and with light darkening in anterior part at level of R_s_. Wing median band expanded along cell r_4+5_ in the direction of vein r-m. Abdomen entirely black, shining.

#### Strongylophthalmyia fascipennis

Taxon classificationAnimaliaDipteraStrongylophthalmyiidae

Frey

Strongylophthalmyia fascipennis Frey, 1928: 102 (male)

##### Material.

1 male, Vietnam, Lai Châu Province, Hoáng Liên (22.33788°N, 103.77922°E), 2068 m, 1 and 7.V. 2013 (T.V. Galinskaya). ZMUM.

#### Strongylophthalmyia metatarsata

Taxon classificationAnimaliaDipteraStrongylophthalmyiidae

Meijere

Strongylophthalmyia metatarsata Meijere, 1919: 35 (female)

##### Material.

1 male, Vietnam, Lai Châu Province, (22.347948°N, 103.769714°E), 1947 m, 22.V.2014 (D. Gavryushin). ZMUM.

#### Strongylophthalmyia splendida

Taxon classificationAnimaliaDipteraStrongylophthalmyiidae

Yang & Wang

Figure 9

Strongylophthalmyia splendida Yang & Wang, 1998 (1996): 459 (female).

##### Material.

1 male, Vietnam, Lai Châu Province, Hoáng Liên (22.347948°N, 103.769714°E), 1700 m, 22.V.2014 (A.L. Ozerov); 1 female, Vietnam, Lai Cai Province, So Pa (22.34147°N, 103.85818°E), 1490 m, 24.X.2015 (D. Gavryushin).

##### Diagnosis.

*Strongylophthalmyia splendida* was described based on single female from Tibet (China), caught at a height of 2050 m. This species is characterized by some interesting and unique characters, including a head that is nearly 1.4 times longer than high (which is caused mainly by lengthening of the occiput). In other *Strongylophthalmyia*, the head is globular (approximately as high as long) and the occiput part is short, only 0.1–0.2 times shorter than length of the eye. Furthermore, the outer vertical seta is absent, the frontal setae are absent and only one short hair-like orbital seta is developed. We also note yellow coloration of frons with a brown round spot between the ocellar tubercle and the vertex.

Other interesting character of the species is the presence of a large seta situated on the anterior part of the mesonotum near the postpronotum in front of posthumeral line (Fig. 9b). Vein R_4+5_ is slightly arcuate, terminating behind the wing apex (Fig. 9a); vein CuA_2_ is more or less straight; cell bm is rather long, terminating approximately at the level of the costal break; cell bm is shorter than the discal cell by approximately in 0.8 times.

##### Description of male.

*Head* yellow, shining in black area, 1.4 times longer than height. frons with brownish to blackish (in view from different angles) rounded spot not reaching eye that extends from level of hind ocelli to postocellar setae. Ocellar tubercle slightly shifted anteriorly: ratio between height of frons from its anterior margin to hind ocelli and from hind ocelli to vertex or vti equal to 1.4. Occiput convex. Face yellow, with row of short setulae along suture. Parafacial covered with silvery grey tomentum. Antenna yellow. First flagellomere rounded, length almost equal to height, very large, 1.9 times less than eye length, with short yellow dorsal setulae. Arista dark brown, basally yellow, bare. Palpus yellow. Chaetotaxy: One short orbital seta, 1 ocellar seta, 1 postocellar seta, 1 inner vertical seta, outer vertical seta absent; frontal setae absent.

*Thorax* black. Postpronotum laterally and dorsally, propleuron, anepisternum around spiracle, basisternum yellow. Mesonotum matte, covered with short yellowish setulae; pleuron shining. Scutellum bare matte. ***Legs*** yellow, mid and hind femora with narrow dark brown apical ring; mid tibia with brownish stroke in basic quarter; hind tibia darkened, yellowish basally and apically. Fore femur dorsally with row of 10 black setae. Fore femur laterally with row of long dense yellowish setulae situated apically aside of femoral apex; basally these setulae 1.3 times longer than those occurring apically. *Wings* greyish, without apical spot and without median transverse band. Vein R_2+3_ short, section of C between R_1_ and R_2+3_ equal to following section (between R_2+3_ and R_4+5_). R_4+5_ and M_1+2_ convergent and almost parallel apically. Section of M_1+2_ between r-m and dm-cu 2.2 times longer than proximal section and 0.45 times shorter than distal section. Calypter brownish grey with 4 long dark setulae on margin. Halter with yellow stem and whitish knob. Chaetotaxy: postpronotal seta absent, 1 + 2 dorsocentral setae, two notopleural setae, one supraalar seta, one postalar seta, one large anepisternal seta, one stout apical scutellar seta. All setae black. Mesonotum with large seta anteriorly, near postpronotum (Fig. 9b: marked by arrow), which we consider as sublateral (Hennig 1973: 184, fig. 109).

*Abdominal* tergites black, shining; tergite 6 brownish yellow; sternites yellow.

Body length 3.4 mm; wing length 3.7 mm.

#### Strongylophthalmyia thaii

Taxon classificationAnimaliaDipteraStrongylophthalmyiidae

L. Papp

Figure 10

Strongylophthalmyia thaii L. Papp, 2006: 171 (male)

##### Material.

2 females, Vietnam, Lai Cai Province, So Pa (22.34147°N, 103.85818°E), 1490 m, 28.X.2015 (D. Gavryushin); 1 male, Thailand, Chang Mai, Sop Poeng (19.122°N, 98.805°E), 13–17. XI. 2009 (N. Vikhrev).

##### Description of female.

*Head* rounded, its length equal to its height. Frons shiny black, with yellowish spot between antenna and eye. Face yellow with row of short blackish setulae along suture. Clypeus black, shining. Parafacial yellowish, covered with silvery grey tomentum. Antenna yellow, first flagellomere transversal, its length 0.7 times shorter than its height, yellowish-orange, widely dark brown on apical margin on outer surface, with short yellow dorsal setulae. Arista dark brown, bare. Mouthparts dark brown; palpus black, yellowish on basal fourth, with short thick black setulae apically. 3 orbital setae, 1 ocellar seta, 1 postocellar seta, 1 inner vertical seta, 1 outer vertical seta; 2 short frontal setae.

*Thorax*. Postpronotum laterally, propleuron, anepisternum around of spiracle and basisternum yellow. Mesonotum black shining, sparsely clothed with short yellowish setulae; pleuron shiny, anepisternum medially without fan of 14–15 very long yellow setulae peculiar to male; scutellum shiny black. *Legs* yellow, hind femora with dark brown narrow apical ring; mid tibia darkened slightly in basal half; hind tibia darkened dorsally and ventrally in basal two thirds. *Wings* transparent. R_2+3_ long: section of C between R_1_ and R_2+3_ 1.6 times longer than a projection of the following section (between R_2+3_ and R_4+5_). R_4+5_ and M_1+2_ nearly parallel apically. Basal section of M_1+2_ between r-m and dm-cu equal to previous section and nearly 2.0 times less than ultimate section. Cell bm approximately 0.5 times shorter than discal cell. Calypter light grey with fan of very long light setulae on margin. Halteres with yellow stem and whitish knob. Chaetotaxy: two small postpronotal seta, two dorsocentral seta, two notopleural setae, one supraalar seta, one postalar seta, one anepisternal seta, one stout apical scutellar seta and one short setae in front of apical ones. All setae black.

*Abdomen* shiny black, tergite 1 and partly 2 yellowish.

Body length 3.9 mm; wing length 3.5 mm.

## Supplementary Material

XML Treatment for Strongylophthalmyia annulipes

XML Treatment for Strongylophthalmyia basisterna

XML Treatment for Strongylophthalmyia dichroa

XML Treatment for Strongylophthalmyia gavryushini

XML Treatment for Strongylophthalmyia obtecta

XML Treatment for Strongylophthalmyia orchidanthae

XML Treatment for Strongylophthalmyia stricta

XML Treatment for Strongylophthalmyia tomentosa

XML Treatment for Strongylophthalmyia angusticollis

XML Treatment for Strongylophthalmyia fascipennis

XML Treatment for Strongylophthalmyia metatarsata

XML Treatment for Strongylophthalmyia splendida

XML Treatment for Strongylophthalmyia thaii
